# 2,4,6-Trinitro­phenyl 4-methyl­benzoate

**DOI:** 10.1107/S1600536812041773

**Published:** 2012-10-13

**Authors:** Rodolfo Moreno-Fuquen, Fabricio Mosquera, Javier Ellena, Juan C. Tenorio, Rodrigo S. Corrêa

**Affiliations:** a Departamento de Química - Facultad de Ciencias, Universidad del Valle, Apartado 25360, Santiago de Cali, Colombia; b Instituto de Física de São Carlos, IFSC, Universidade de São Paulo, USP, São Carlos, SP, Brazil; c Departamento de Química, Universidade Federal de São Carlos, CEP 13565-905, São Carlos, SP, Brazil

## Abstract

In the title compound, C_14_H_9_N_3_O_8_, the benzene rings form a dihedral angle of 69.02 (5)°. The central ester group is rotated by 25.86 (9)° relative to the *p*-tolyl group. In the crystal, the mol­ecules are linked by C—H⋯O inter­actions into helical chains along [010].

## Related literature

For optical, pharmacological and crystalline properties of picric acid, see: Khan *et al.* (2010 ▶); Zaderenko *et al.* (1997 ▶). For picric acid derivatives, see: Bertolasi *et al.* (2011 ▶). For bond-length data, see: Allen *et al.* (1987 ▶). For similar structures, see: Moreno-Fuquen *et al.* (2012 ▶); Bibi *et al.* (2009 ▶); Shibakami *et al.* (1994 ▶); Shibakami & Sekiya (1995 ▶); For hydrogen bonding, see: Nardelli (1995 ▶) and for supra­molecular aggregation behaviour of isomers, see: Glidewell *et al.* (2005 ▶).
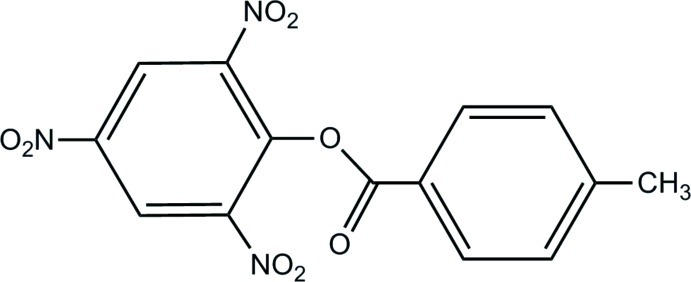


## Experimental

### 

#### Crystal data

C_14_H_9_N_3_O_8_
*M* *_r_* = 347.24
Monoclinic, 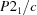

*a* = 7.6126 (2) Å
*b* = 8.2124 (2) Å
*c* = 23.9893 (7) Å
β = 94.448 (1)°
*V* = 1495.24 (7) Å^3^
*Z* = 4
Mo *K*α radiation
μ = 0.13 mm^−1^
*T* = 295 K
0.27 × 0.22 × 0.18 mm

#### Data collection

Nonius KappaCCD diffractometer
5494 measured reflections
3044 independent reflections
2296 reflections with *I* > 2σ(*I*)
*R*_int_ = 0.017

#### Refinement

*R*[*F* ^2^ > 2σ(*F* ^2^)] = 0.043
*wR*(*F* ^2^) = 0.123
*S* = 1.02
3044 reflections
227 parameters
1 restraint
H-atom parameters constrained
Δρ_max_ = 0.18 e Å^−3^
Δρ_min_ = −0.24 e Å^−3^

### 

Data collection: *COLLECT* (Nonius, 2000 ▶); cell refinement: *HKL* *SCALEPACK* (Otwinowski & Minor, 1997 ▶); data reduction: *HKL* *DENZO* (Otwinowski & Minor, 1997 ▶) and *SCALEPACK*; program(s) used to solve structure: *SHELXS97* (Sheldrick, 2008 ▶); program(s) used to refine structure: *SHELXL97* (Sheldrick, 2008 ▶); molecular graphics: *ORTEP-3 for Windows* (Farrugia, 1997 ▶) and *Mercury* (Macrae *et al.*, 2006 ▶); software used to prepare material for publication: *WinGX* (Farrugia, 1999 ▶).

## Supplementary Material

Crystal structure: contains datablock(s) I, global. DOI: 10.1107/S1600536812041773/ld2074sup1.cif

Structure factors: contains datablock(s) I. DOI: 10.1107/S1600536812041773/ld2074Isup2.hkl

Supplementary material file. DOI: 10.1107/S1600536812041773/ld2074Isup3.cml

Additional supplementary materials:  crystallographic information; 3D view; checkCIF report

## Figures and Tables

**Table 1:** Hydrogen-bond geometry (Å, °)

*D*—H⋯*A*	*D*—H	H⋯*A*	*D*⋯*A*	*D*—H⋯*A*
C3—H3⋯O8^i^	0.93	2.50	3.4286 (19)	175

Symmetry code: (i) 
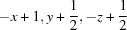
.

**Fig. 1.**
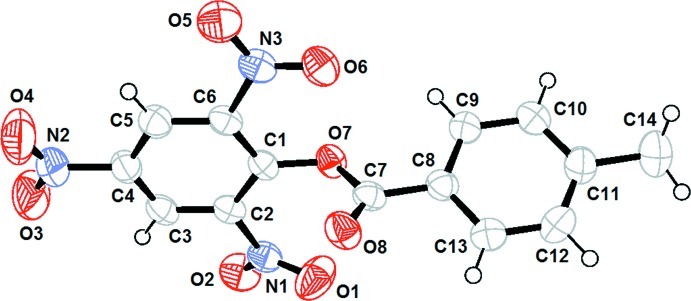
Molecular conformation and atom numbering scheme of the title compound with displacement ellipsoids drawn at 50% probability level. H atoms are shown as spheres of arbitrary radius.

**Fig. 2.**
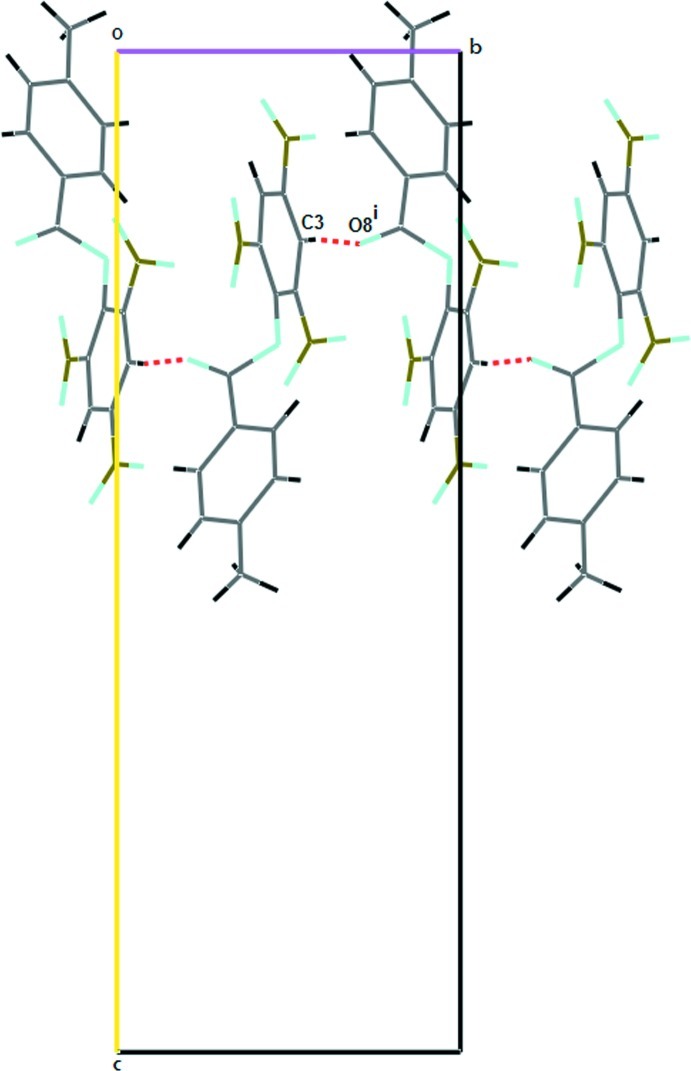
Part of the crystal structure of (I) showing the formation of helical chains along [010]. Symmetry code: (i) -x+1,+y+1/2,-z+1/2.

## supplementary crystallographic information

### Comment

The title compound, C_14_H_9_O_8_N_3_ [2,4,6-trinitrophenyl 4-methylbenzoate] (I), belongs to a group of molecules known as nitro aryl benzoates. Recently, we have been investigating the synthesis, properties and main features of these nitroaromatic compounds, in particular - the derivatives of picric acid (trinitrophenol - TNP) (Bertolasi *et al.*, 2011). These compounds have never been synthesized before and there is no any prior entries in CCSD related to such esters, except 2,4,6-trinitrophenyl 3-methylbenzoate (TNP3MeBA), an isomer of the title compound, that we recently published (Moreno-Fuquen *et al.*, 2012). Our special interest in TNP is attributable to the optical, pharmacological and solid-state properties of its molecular adducts (Khan *et al.*, 2010); Zaderenko *et al.*, 1997) and the absence of any information of the picric acid as a part of aryl esters that probably can show similar or more interesting properties. In order to find out the molecular conformation of (I) and its supramolecular behavior, the title compound was synthesized. The molecular structure of (I) is presented on Fig. 1 with the numbering scheme similar to that for TNP-3MeBA in order to simplify structural comparisons. In the title structure, the phenolic C1—O7 bond length is significantly shortened [1.3715 (17) Å] and the benzoic C7—O7 bond length is significantly elongated [1.3898 (17) Å] as compared to other phenylbenzoates systems [1.422 (5) Å and 1.354 (5) Å respectively (Shibakami *et al.*, 1994) and 1.415 (2) Å and 1.351 (2) Å respectively (Shibakami & Sekiya, 1995)]. However, these parameters are pretty close to the values presented in other crystal systems which contain similar substituents with respect to this structure, more specifically when there is nitro substituent in any of the benzene rings at the benzoate [for C1—O7= 1.395 (2) Å and for C7—O7= 1.376 (3) Å (Bibi *et al.*, 2009) and 1.3676 (17) Å and 1.3820 (18) Å respectively (Moreno-Fuquen *et al.*, 2012)]. Other bond lengths and bond angles of (I) agree with the literature values (Allen *et al.*, 1987). The benzene rings of (I) form a dihedral angle of 69.02 (5)°, compared with values of 87.48 (5)° in TNP3MeBA. The central ester moiety forms an angle of 25.86 (9)° with the methylbenzene ring to which it is attached. This value is similar to the corresponding angle in its structural isomer TNP3MeBA - 19.42 (7)°. The nitro groups form dihedral angles with the adjacent benzene ring of 30.57 (11)°, 14.75 (16)° and 7.37 (17)° for O1—N1—O2, O3—N2—O4 and O5—N3—O6, respectively. The molecules are packed forming weak interactions C—H···O in one-dimensional helical chains which grow along [010] (see Fig. 2). The C3 atom of the phenyl ring at (*x*,*y*,*z*) acts as a hydrogen-bond donor to carbonyl atom O8 at (-*x* + 1,+*y* + 1/2,-*z* + 1/2) (see Table 1; Nardelli, 1995). We have found that (I) and its isomer TNP3MeBA show a marked similarity in terms of spatial group, unit-cell parameters and finally the intermolecular interactions given the supramolecular aggregation of these isomers. This behavior is very uncommon in compounds showing constitutional isomerism, since small changes in the molecular structures of the isomers usually lead to large changes in molecular aggregation of the structures (Glidewell *et al.*, 2005).

### Experimental

The reagents and solvents for the synthesis were obtained from the Aldrich Chemical Co., and were used without additional purification. The title molecule was obtained through a two-step reaction. First the 4-methylbenzoic acid (0.25 g, 1.836 mmol) was refluxed in an excess of thionyl chloride (10 ml) during an hour. Then thionyl chloride was distilled off under reduced pressure to purify the 4-methylbenzoyl chloride obtained as a pale yellow traslucent liquid. The same reaction flask was rearranged and a solution of picric acid (0.42 g, 1.835 mmol) in acetonitrile was added dropwise with constant stirring. The reaction mixture was let to reflux for about an hour. A pale yellow solid was obtained after leaving the solvent to evaporate. The solid was washed with distilled water and cold methanol to eliminate impurities. Crystals of good quality and suitable for single-crystal X-ray diffraction were grown from acetonitrile. IR spectra were recorded on a FT—IR SHIMADZU IR-Affinity-1 spectrophotometer. Pale Yellow crystals; yield 38%; m.p 417 (1) K. IR (KBr) 3112.86 cm^-1^, 3086.07 cm^-1^ (aromatic C—H); 2962.07 cm^-1^, 2915.70 cm^-1^ (methyl C—H); 1754.68 cm^-1^ (ester C=O); 1609.43 cm^-1^ (C=C); 1549.55 cm^-1^, 1341.69 cm^-1^ (–NO2); 1224.07 cm^-1^ (C(=O)—O).

### Refinement

All the H-atoms attached to C atoms were positioned at geometrically idealized positions and treated as riding with C—H= 0.93 Å (aromatic) and 0.96 Å (methyl) with *U*_iso_(H) = 1.2 *U*_eq_ (aromatic) and *U*_iso_(H) = 1.5 *U*_eq_ (methyl). The positions of H atoms of the methyl group were rotationally optimized

### Crystal data

**Table:**

C_14_H_9_N_3_O_8_	*F*(000) = 712
*M**_r_* = 347.24	*D*_x_ = 1.543 Mg m^−^^3^
Monoclinic, *P*2_1_/*c*	Melting point: 417(1) K
Hall symbol: -P 2ybc	Mo *K*α radiation, λ = 0.71073 Å
*a* = 7.6126 (2) Å	Cell parameters from 3076 reflections
*b* = 8.2124 (2) Å	θ = 3.0–26.4°
*c* = 23.9893 (7) Å	µ = 0.13 mm^−^^1^
β = 94.448 (1)°	*T* = 295 K
*V* = 1495.24 (7) Å^3^	Block, pale-yellow
*Z* = 4	0.27 × 0.22 × 0.18 mm

### Data collection

**Table:**

Nonius KappaCCD diffractometer	2296 reflections with *I* > 2σ(*I*)
Radiation source: fine-focus sealed tube	*R*_int_ = 0.017
Graphite monochromator	θ_max_ = 26.4°, θ_min_ = 3.0°
CCD rotation images, thick slices scans	*h* = −9→9
5494 measured reflections	*k* = −9→10
3044 independent reflections	*l* = −29→29

### Refinement

**Table:**

Refinement on *F*^2^	Primary atom site location: structure-invariant direct methods
Least-squares matrix: full	Secondary atom site location: difference Fourier map
*R*[*F*^2^ > 2σ(*F*^2^)] = 0.043	Hydrogen site location: inferred from neighbouring sites
*wR*(*F*^2^) = 0.123	H-atom parameters constrained
*S* = 1.02	*w* = 1/[σ^2^(*F*_o_^2^) + (0.0698*P*)^2^ + 0.224*P*] where *P* = (*F*_o_^2^ + 2*F*_c_^2^)/3
3044 reflections	(Δ/σ)_max_ < 0.001
227 parameters	Δρ_max_ = 0.18 e Å^−^^3^
1 restraint	Δρ_min_ = −0.24 e Å^−^^3^

### Special details

**Table:**

Geometry. All esds (except the esd in the dihedral angle between two l.s. planes) are estimated using the full covariance matrix. The cell esds are taken into account individually in the estimation of esds in distances, angles and torsion angles; correlations between esds in cell parameters are only used when they are defined by crystal symmetry. An approximate (isotropic) treatment of cell esds is used for estimating esds involving l.s. planes.
Refinement. Refinement of *F*^2^ against ALL reflections. The weighted *R*-factor *wR* and goodness of fit *S* are based on *F*^2^, conventional *R*-factors *R* are based on *F*, with *F* set to zero for negative *F*^2^. The threshold expression of *F*^2^ > σ(*F*^2^) is used only for calculating *R*-factors(gt) *etc*. and is not relevant to the choice of reflections for refinement. *R*-factors based on *F*^2^ are statistically about twice as large as those based on *F*, and *R*- factors based on ALL data will be even larger.

### Fractional atomic coordinates and isotropic or equivalent isotropic displacement parameters (Å^2^)

**Table:**

	*x*	*y*	*z*	*U*_iso_*/*U*_eq_	
O1	0.4307 (2)	0.4914 (2)	0.33344 (6)	0.0840 (5)	
O2	0.56533 (16)	0.66653 (15)	0.28637 (6)	0.0633 (4)	
O3	0.5292 (2)	0.5772 (3)	0.08518 (7)	0.1011 (6)	
O4	0.3254 (2)	0.4222 (3)	0.04835 (7)	0.1007 (6)	
O5	−0.18224 (17)	0.33867 (18)	0.14637 (6)	0.0732 (4)	
O6	−0.17197 (15)	0.33696 (15)	0.23563 (5)	0.0595 (3)	
O7	0.09683 (13)	0.46979 (12)	0.29293 (4)	0.0407 (3)	
O8	0.16920 (15)	0.20662 (13)	0.30759 (5)	0.0525 (3)	
N1	0.45379 (16)	0.56271 (17)	0.29052 (6)	0.0469 (3)	
N2	0.3987 (2)	0.4953 (2)	0.08753 (7)	0.0664 (4)	
N3	−0.10536 (17)	0.35918 (15)	0.19191 (6)	0.0460 (3)	
C1	0.17239 (18)	0.46265 (16)	0.24291 (6)	0.0362 (3)	
C2	0.34413 (18)	0.51938 (17)	0.23953 (6)	0.0386 (3)	
C3	0.41940 (19)	0.53543 (18)	0.18945 (7)	0.0433 (4)	
H3	0.5318	0.5787	0.1878	0.052*	
C4	0.3213 (2)	0.48477 (19)	0.14189 (7)	0.0462 (4)	
C5	0.1537 (2)	0.42336 (19)	0.14288 (7)	0.0457 (4)	
H5	0.0918	0.3878	0.1102	0.055*	
C6	0.07943 (18)	0.41560 (17)	0.19332 (6)	0.0393 (3)	
C7	0.09911 (18)	0.32603 (18)	0.32354 (7)	0.0396 (4)	
C8	0.00660 (19)	0.34449 (18)	0.37471 (7)	0.0420 (4)	
C9	−0.1286 (2)	0.4560 (2)	0.37848 (7)	0.0516 (4)	
H9	−0.1580	0.5273	0.3491	0.062*	
C10	−0.2198 (2)	0.4607 (2)	0.42623 (8)	0.0618 (5)	
H10	−0.3118	0.5344	0.4283	0.074*	
C11	−0.1768 (2)	0.3578 (3)	0.47078 (7)	0.0595 (5)	
C12	−0.0394 (2)	0.2490 (2)	0.46659 (7)	0.0617 (5)	
H12	−0.0074	0.1803	0.4965	0.074*	
C13	0.0511 (2)	0.2402 (2)	0.41905 (7)	0.0537 (4)	
H13	0.1414	0.1648	0.4167	0.064*	
C14	−0.2789 (3)	0.3625 (4)	0.52240 (9)	0.0880 (7)	
H14A	−0.2015	0.3370	0.5547	0.132*	
H14B	−0.3727	0.2841	0.5187	0.132*	
H14C	−0.3274	0.4693	0.5265	0.132*	

### Atomic displacement parameters (Å^2^)

**Table:**

	*U*^11^	*U*^22^	*U*^33^	*U*^12^	*U*^13^	*U*^23^
O1	0.0738 (9)	0.1154 (12)	0.0599 (8)	−0.0385 (9)	−0.0135 (7)	0.0239 (8)
O2	0.0497 (7)	0.0579 (8)	0.0813 (9)	−0.0212 (6)	−0.0008 (6)	−0.0053 (6)
O3	0.0687 (10)	0.1650 (17)	0.0728 (10)	−0.0334 (11)	0.0253 (8)	0.0139 (11)
O4	0.0954 (12)	0.1528 (17)	0.0567 (9)	−0.0247 (12)	0.0243 (8)	−0.0252 (10)
O5	0.0516 (7)	0.0986 (11)	0.0677 (9)	−0.0220 (7)	−0.0064 (6)	−0.0147 (7)
O6	0.0435 (6)	0.0694 (8)	0.0667 (8)	−0.0163 (6)	0.0113 (6)	0.0084 (6)
O7	0.0426 (6)	0.0342 (5)	0.0468 (6)	−0.0005 (4)	0.0123 (5)	0.0012 (4)
O8	0.0546 (7)	0.0402 (6)	0.0648 (8)	0.0080 (5)	0.0179 (6)	0.0051 (5)
N1	0.0364 (7)	0.0469 (8)	0.0571 (8)	−0.0044 (6)	0.0021 (5)	−0.0011 (6)
N2	0.0529 (9)	0.0924 (12)	0.0560 (10)	−0.0001 (9)	0.0169 (8)	0.0064 (9)
N3	0.0384 (7)	0.0389 (7)	0.0608 (9)	−0.0058 (5)	0.0028 (7)	−0.0017 (6)
C1	0.0359 (7)	0.0275 (7)	0.0461 (8)	0.0019 (5)	0.0083 (6)	0.0008 (6)
C2	0.0350 (7)	0.0306 (7)	0.0504 (8)	0.0001 (6)	0.0036 (6)	0.0003 (6)
C3	0.0338 (7)	0.0396 (8)	0.0576 (10)	−0.0003 (6)	0.0103 (7)	0.0051 (7)
C4	0.0440 (8)	0.0482 (9)	0.0478 (9)	0.0046 (7)	0.0117 (7)	0.0044 (7)
C5	0.0443 (8)	0.0447 (8)	0.0481 (9)	0.0007 (7)	0.0036 (7)	−0.0014 (7)
C6	0.0334 (7)	0.0331 (7)	0.0515 (9)	−0.0006 (6)	0.0038 (6)	0.0007 (6)
C7	0.0318 (7)	0.0368 (8)	0.0503 (9)	−0.0017 (6)	0.0035 (6)	0.0033 (7)
C8	0.0362 (7)	0.0431 (8)	0.0469 (9)	−0.0026 (6)	0.0037 (6)	0.0027 (7)
C9	0.0464 (9)	0.0600 (10)	0.0492 (10)	0.0113 (8)	0.0083 (7)	0.0104 (8)
C10	0.0504 (10)	0.0798 (13)	0.0566 (11)	0.0186 (9)	0.0125 (8)	0.0066 (9)
C11	0.0529 (10)	0.0821 (13)	0.0440 (10)	−0.0031 (9)	0.0074 (8)	0.0034 (9)
C12	0.0652 (11)	0.0722 (12)	0.0472 (10)	0.0042 (10)	0.0009 (8)	0.0161 (9)
C13	0.0493 (9)	0.0575 (10)	0.0542 (10)	0.0091 (8)	0.0031 (8)	0.0082 (8)
C14	0.0818 (15)	0.130 (2)	0.0552 (13)	0.0046 (14)	0.0247 (11)	0.0093 (13)

### Geometric parameters (Å, º)

**Table:**

O1—N1	1.2089 (18)	C5—C6	1.376 (2)
O2—N1	1.2128 (17)	C5—H5	0.9300
O3—N2	1.204 (2)	C7—C8	1.470 (2)
O4—N2	1.214 (2)	C8—C9	1.386 (2)
O5—N3	1.2106 (18)	C8—C13	1.387 (2)
O6—N3	1.2135 (17)	C9—C10	1.385 (2)
O7—C1	1.3715 (17)	C9—H9	0.9300
O7—C7	1.3898 (17)	C10—C11	1.382 (3)
O8—C7	1.1936 (18)	C10—H10	0.9300
N1—C2	1.470 (2)	C11—C12	1.385 (3)
N2—C4	1.474 (2)	C11—C14	1.513 (2)
N3—C6	1.4790 (19)	C12—C13	1.379 (2)
C1—C6	1.391 (2)	C12—H12	0.9300
C1—C2	1.396 (2)	C13—H13	0.9300
C2—C3	1.377 (2)	C14—H14A	0.9600
C3—C4	1.378 (2)	C14—H14B	0.9600
C3—H3	0.9300	C14—H14C	0.9600
C4—C5	1.374 (2)		
			
C1—O7—C7	115.91 (11)	O8—C7—O7	121.07 (13)
O1—N1—O2	124.00 (14)	O8—C7—C8	127.42 (14)
O1—N1—C2	118.73 (13)	O7—C7—C8	111.50 (12)
O2—N1—C2	117.24 (13)	C9—C8—C13	119.77 (15)
O3—N2—O4	124.83 (17)	C9—C8—C7	121.94 (14)
O3—N2—C4	117.50 (17)	C13—C8—C7	118.19 (14)
O4—N2—C4	117.66 (16)	C10—C9—C8	119.65 (15)
O5—N3—O6	123.63 (13)	C10—C9—H9	120.2
O5—N3—C6	117.16 (13)	C8—C9—H9	120.2
O6—N3—C6	119.20 (13)	C11—C10—C9	121.23 (16)
O7—C1—C6	122.69 (12)	C11—C10—H10	119.4
O7—C1—C2	119.66 (13)	C9—C10—H10	119.4
C6—C1—C2	117.35 (13)	C10—C11—C12	118.26 (16)
C3—C2—C1	122.52 (14)	C10—C11—C14	120.74 (18)
C3—C2—N1	117.06 (13)	C12—C11—C14	120.99 (18)
C1—C2—N1	120.41 (13)	C13—C12—C11	121.48 (16)
C2—C3—C4	117.26 (14)	C13—C12—H12	119.3
C2—C3—H3	121.4	C11—C12—H12	119.3
C4—C3—H3	121.4	C12—C13—C8	119.59 (16)
C5—C4—C3	122.72 (15)	C12—C13—H13	120.2
C5—C4—N2	118.18 (15)	C8—C13—H13	120.2
C3—C4—N2	119.10 (15)	C11—C14—H14A	109.5
C4—C5—C6	118.54 (15)	C11—C14—H14B	109.5
C4—C5—H5	120.7	H14A—C14—H14B	109.5
C6—C5—H5	120.7	C11—C14—H14C	109.5
C5—C6—C1	121.51 (13)	H14A—C14—H14C	109.5
C5—C6—N3	116.71 (13)	H14B—C14—H14C	109.5
C1—C6—N3	121.76 (13)		
			
C7—O7—C1—C6	87.32 (16)	C2—C1—C6—C5	0.5 (2)
C7—O7—C1—C2	−99.17 (15)	O7—C1—C6—N3	−4.2 (2)
O7—C1—C2—C3	−171.34 (12)	C2—C1—C6—N3	−177.83 (12)
C6—C1—C2—C3	2.5 (2)	O5—N3—C6—C5	−6.7 (2)
O7—C1—C2—N1	9.53 (19)	O6—N3—C6—C5	173.77 (13)
C6—C1—C2—N1	−176.61 (12)	O5—N3—C6—C1	171.70 (14)
O1—N1—C2—C3	−148.89 (16)	O6—N3—C6—C1	−7.9 (2)
O2—N1—C2—C3	29.57 (19)	C1—O7—C7—O8	2.8 (2)
O1—N1—C2—C1	30.3 (2)	C1—O7—C7—C8	−176.87 (12)
O2—N1—C2—C1	−151.26 (14)	O8—C7—C8—C9	−151.99 (17)
C1—C2—C3—C4	−3.3 (2)	O7—C7—C8—C9	27.6 (2)
N1—C2—C3—C4	175.80 (13)	O8—C7—C8—C13	24.5 (2)
C2—C3—C4—C5	1.3 (2)	O7—C7—C8—C13	−155.96 (14)
C2—C3—C4—N2	−178.25 (13)	C13—C8—C9—C10	−0.9 (3)
O3—N2—C4—C5	165.82 (18)	C7—C8—C9—C10	175.47 (16)
O4—N2—C4—C5	−13.5 (3)	C8—C9—C10—C11	1.1 (3)
O3—N2—C4—C3	−14.6 (3)	C9—C10—C11—C12	0.0 (3)
O4—N2—C4—C3	166.10 (18)	C9—C10—C11—C14	−179.2 (2)
C3—C4—C5—C6	1.5 (2)	C10—C11—C12—C13	−1.2 (3)
N2—C4—C5—C6	−178.95 (14)	C14—C11—C12—C13	178.0 (2)
C4—C5—C6—C1	−2.4 (2)	C11—C12—C13—C8	1.3 (3)
C4—C5—C6—N3	175.99 (13)	C9—C8—C13—C12	−0.2 (3)
O7—C1—C6—C5	174.10 (13)	C7—C8—C13—C12	−176.76 (16)

### Hydrogen-bond geometry (Å, º)

**Table:**

*D*—H···*A*	*D*—H	H···*A*	*D*···*A*	*D*—H···*A*
C3—H3···O8^i^	0.93	2.50	3.4286 (19)	175

Symmetry code: (i) −*x*+1, *y*+1/2, −*z*+1/2.
